# Calcium and Albumin Blood Tests, Ethnicity, and Cancer Incidence in Primary Care in the UK

**DOI:** 10.3390/cancers17172913

**Published:** 2025-09-05

**Authors:** Liz Down, Melissa Barlow, Luke T. A. Mounce, Jessica Watson, Samuel W. D. Merriel, Sarah E. R. Bailey, Tanimola Martins

**Affiliations:** 1 Department of Health and Community Sciences, University of Exeter, Exeter EX1 2LU, UK; m.barlow@exeter.ac.uk (M.B.); l.t.a.mounce@exeter.ac.uk (L.T.A.M.); s.e.r.bailey@exeter.ac.uk (S.E.R.B.); tom207@exeter.ac.uk (T.M.); 2 Centre for Academic Primary Care (CAPC), University of Bristol, Bristol BS8 1TH, UK; jessica.watson@bristol.ac.uk; 3 Centre for Primary Care & Health Services Research, University of Manchester, Manchester M13 9QQ, UK; samuel.merriel@manchester.ac.uk

**Keywords:** serum albumin, hypercalcemia, early detection of cancer, ethnicity, primary health care

## Abstract

**Objectives:** This study aimed to assess any ethnic differences in blood calcium and albumin levels for patients receiving these tests in primary care, and to investigate how this affects the use of these markers in assessing cancer risk. **Methods:** The analysis was based on a primary care dataset comprising patients in England. Multilevel logistic regression was used to investigate the relationship between blood test results and cancer risk by ethnic group. **Results:** A total of 4,632,856 patients were eligible for the albumin analysis, and 1,979,763 for the calcium analysis. Raised calcium levels were indicative of an increased risk of cancer, with diagnostic odds ratios (ORs) ranging from 2.0 to 2.7 for the different ethnic groups. ORs for myeloma were between 6.6 and 13.6. Similarly, low albumin was associated with an increased risk of cancer with an OR of between 3.2 and 3.8, myeloma (OR between 8.7 and 10.0), and liver cancer (OR between 9.2 and 15.7). **Conclusions:** Albumin and corrected calcium were effective indicators of cancer risk, and more specifically of risk of myeloma. Albumin levels were also linked with liver cancer risk. While there are some differences in typical corrected calcium and albumin levels between ethnic groups, there was no evidence that this had an effect on the usefulness of these markers to infer cancer risk.

## Simple Summary

For many common blood tests, typical values differ for patients from different ethnic groups. Although it is known that albumin and calcium tests may be useful in identifying patients with a higher-than-average cancer risk, the evidence is limited and does not take into account patient ethnicity. Examining the blood test results in a large English primary care dataset demonstrated that having either low albumin or high calcium was predictive of cancer risk, and more specifically risk of myeloma. Having low albumin was also predictive of liver cancer. There were no differences in how effective these blood tests are at estimating cancer risk for patients from different ethnic groups.

## 1. Introduction

Asian and Black patients are more likely to be diagnosed at an advanced stage of certain cancer types compared to their White counterparts in the UK [1]. This disparity is particularly concerning because advanced stage diagnosis significantly reduces treatment options and survival outcomes. Two major initiatives in the NHS Long Term Plan are to reduce the proportion of advanced stage diagnoses to 25% from around 50%, while also taking steps to reduce ethnic inequalities in cancer survival [2].

Blood tests are often a first-line investigation for patients presenting with both cancer-specific and non-specific symptoms and are readily available to primary care clinicians. Several primary care blood tests are known to aid cancer risk assessment, including haemoglobin, ferritin, blood glucose, calcium, white cell count, platelet count, PSA, and CA-125 [3]. Recent evidence suggests that the efficacy of haemoglobin level, mean cell volume, and PSA level in detecting cancer may differ between ethnic groups [4,5].

A recent systematic review found some ethnic differences in albumin levels, with White patients typically having higher levels than Black patients, but found no difference in calcium levels [6]. No studies were found assessing calcium levels, ethnicity, and cancer risk. One study in the USA found an inverse association between albumin levels and lung cancer risk for African Americans, but not for European Americans, although the number of patients was small in the latter group [7].

Elevated calcium levels in primary care can indicate increased risk of malignancy, particularly multiple myeloma, as well as other conditions such as hyperparathyroidism [8,9,10,11]. Low albumin concentrations can signal poor nutrition, liver disease, sepsis, or nephrotic syndrome, and have also been associated with an elevated risk of cancer in various populations [7,12,13,14,15,16,17]. However, there are no UK guidelines attributing low albumin levels to specific cancer sites.

This study aimed to investigate the link between abnormal calcium and albumin levels and subsequent cancer diagnosis in patients of different ethnic groups consulting in English primary care.

## 2. Materials and Methods

### 2.1. Data Sources

This English cohort study used routinely collected electronic primary care records from the Clinical Practice Research Datalink (CPRD) Aurum database [18], linked to secondary care records from Hospital Episode Statistics (HES) [19], and the National Cancer Registration and Analysis Service (NCRAS) [20].

### 2.2. Patient Selection

Eligible patients were aged 40 years or over, with no prior cancer diagnosis (except localised skin cancer), a record of ethnicity, with a serum albumin and/or calcium test recorded between 2010 and 2017. If patients had more than one blood test within the study period, the first blood test available in the patients’ record was selected.

### 2.3. Variable Derivation

In order to account for laboratories using different thresholds to define abnormal calcium and albumin levels, the most common laboratory threshold from the dataset was used for each blood test. Normal calcium was defined as levels of between 2.15 mmol/L and 2.6 mmol/L, while normal albumin was between 35 g/L and 50 g/L, closely aligning with clinical guidance [21] and published literature [22]. Corrected calcium levels were used for this analysis, adjusted for albumin levels, as this is typically used in clinical practice [23].

Patient ethnicity was derived from the CPRD Aurum data where possible, with the addition of HES APC data where no ethnicity was available from the primary care data [24,25,26].

Cancer status was determined by the presence of a cancer diagnosis in the NCRAS dataset within one-year of the index blood test date. Advanced cancer was defined as TNM stage T3, T4 or M1.

Demographic covariates included patient sex, age, socioeconomic deprivation status, smoking status, multimorbidity burden, presence of haemoglobinopathies, and body mass index (BMI) category. Age was grouped in 10-year age bands (40 to 49 years, 50 to 59 years, 60 to 69 years, 70 years and above). The measure of socioeconomic deprivation used was the quintile of the rank of a patient’s area-based deprivation score, using IMD2015 [27], a composite measure of social and material deprivation indicators. Multimorbidity burden was calculated using the Cambridge Multimorbidity Score (CMS) methodology [28], and categorised into four levels: no multimorbidity, and three quantiles of multimorbidity severity. An indicator was generated to denote patients with a record of common haemoglobinopathies (sickle cell, thalassaemia variants, and unspecified haemoglobinopathy—including patients with any of these conditions, and carriers). BMI was categorised into underweight (≤18.49 kg/m^2^), normal weight (18.5 to 24.99 kg/m^2^), overweight (25.0 to 29.99 kg/m^2^), obese (≥30.0 kg/m^2^), or not recorded.

### 2.4. Statistical Models

Multilevel logistic regression was used to investigate the relationship between an abnormal blood test result and cancer risk for patients in each ethnic group, clustered by GP practice and adjusted for the covariates described above. An interaction term was included between blood test result and patient ethnicity. The primary outcome measure was one-year cancer incidence, with subsequent cancer site-specific models. Site-specific models comprised myeloma for the calcium cohort, in accordance with UK NICE guidance [3]. Due to the absence of guidelines relating to low albumin levels and site-specific cancer incidence, the sites where one of the three main ethnic groups had diagnostic odds ratios (OR) of 10 or more are included in this report. Additionally, cancer diagnosis at an advanced stage was also assessed.

The marginal distributions of the models were used to obtain one-year cancer incidence, and to generate ORs comparing incidence in those with and without an abnormal blood test result.

Although models only included patients with normal or high calcium, or normal or low albumin, test result distributions in tables and figures include data for patients with any blood test result.

Analyses were conducted using Stata MP version 18.0. Plots were generated using R 4.3.3. “Angel Food Cake”.

### 2.5. Sample Size Calculations

Sample size calculations determined that 1118 patients would be required in each group (ethnicity and test abnormality) to detect a cancer incidence of 3% with a margin of error of <1 percentage point. This sample size was achieved for each of the three main ethnic groups (White, Asian, and Black), but not for the Mixed and Other ethnic groups.

### 2.6. Patient and Public Involvement and Engagement

A Patient and Public Involvement and Engagement group was specifically recruited for this study, ensuring representation from the three main ethnic groups analysed in this study (White, Asian, and Black). Their valuable input informed the discussion and conclusion.

## 3. Results

### 3.1. Cohort

**Figure 1:**
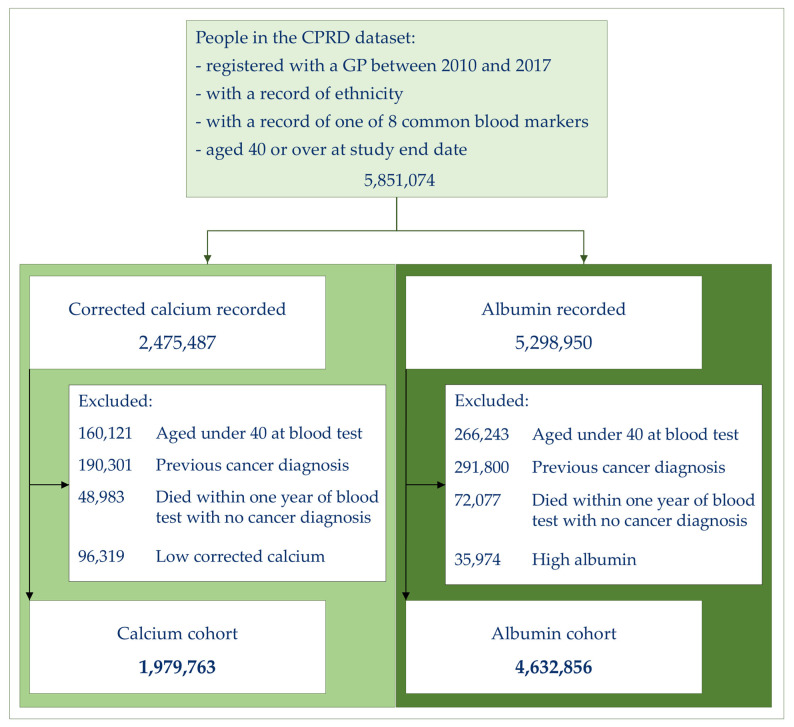
Cohort selection.

The number of patients eligible for this study are illustrated in Figure 1. After exclusion criteria were applied, 1,979,763 patients with a normal or high calcium result were available for analysis. The majority of these patients were White (86%), with 7% Asian, 5% Black, 1% in the Mixed group, and 1% in the Other group (Table 1).

The total number of patients available for the albumin analysis was 4,632,856. The ethnic distribution for the albumin cohort was similar at 87% White, 7% Asian, 4% Black, and with 1% in the Mixed and Other groups (Table 1).

White patients were, on average, older with a median age of 58, compared to 50 for Asian patients, and 49 for all other patients. White patients were more likely to have a higher morbidity burden, and to have a history of smoking compared to the other groups. Black patients were most likely to be overweight or obese, or to live in a deprived area. Black patients had the highest rate of haemoglobinopathy, although patients in the Mixed and Asian group also had relatively high rates (Table 1).

### 3.2. Blood Test Result Distribution

**Figure 2:**
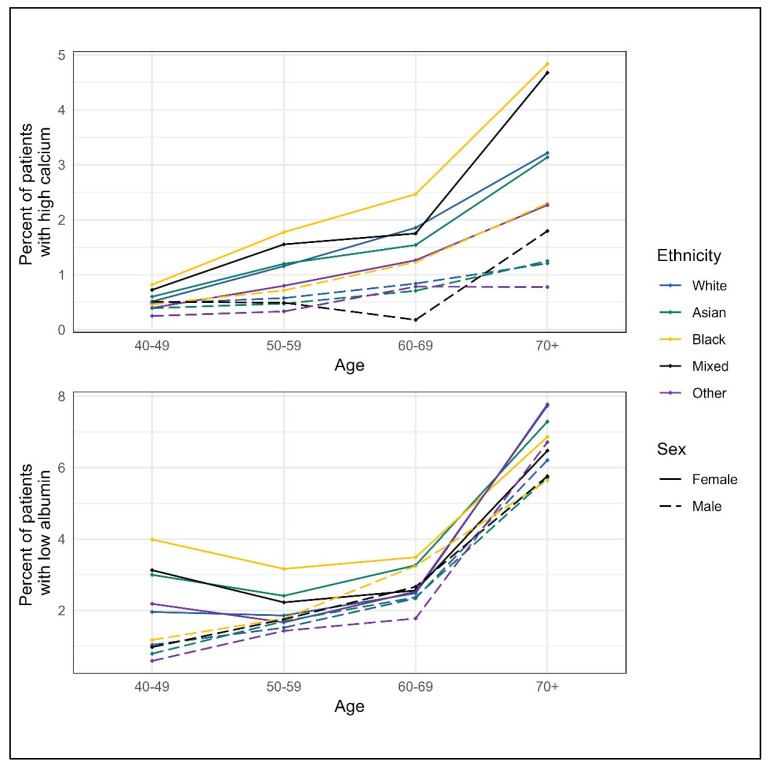
Abnormal blood tests by sex and ethnic group *. * Blood test distribution values are based on the entire eligible cohort for each blood result, including those patients with raised albumin or low corrected calcium.

There was little difference in albumin and calcium blood test values between the ethnic groups (Table 2), although stratification by age and sex suggests the highest proportion of patients with abnormal results may be in Black patients (Figure 2). Calcium values increased with age and were higher for women than for men (Figure 2, Table A1). Albumin levels decreased with age and were lower for women (Figure 2, Table A1).

### 3.3. Cancer Risk by Blood Test Result

The number of patients in the Mixed and Other group were relatively low, leading to effect estimates with very wide confidence intervals, so the results for these groups are not presented here, but can be found in Table A2.

**Figure 3:**
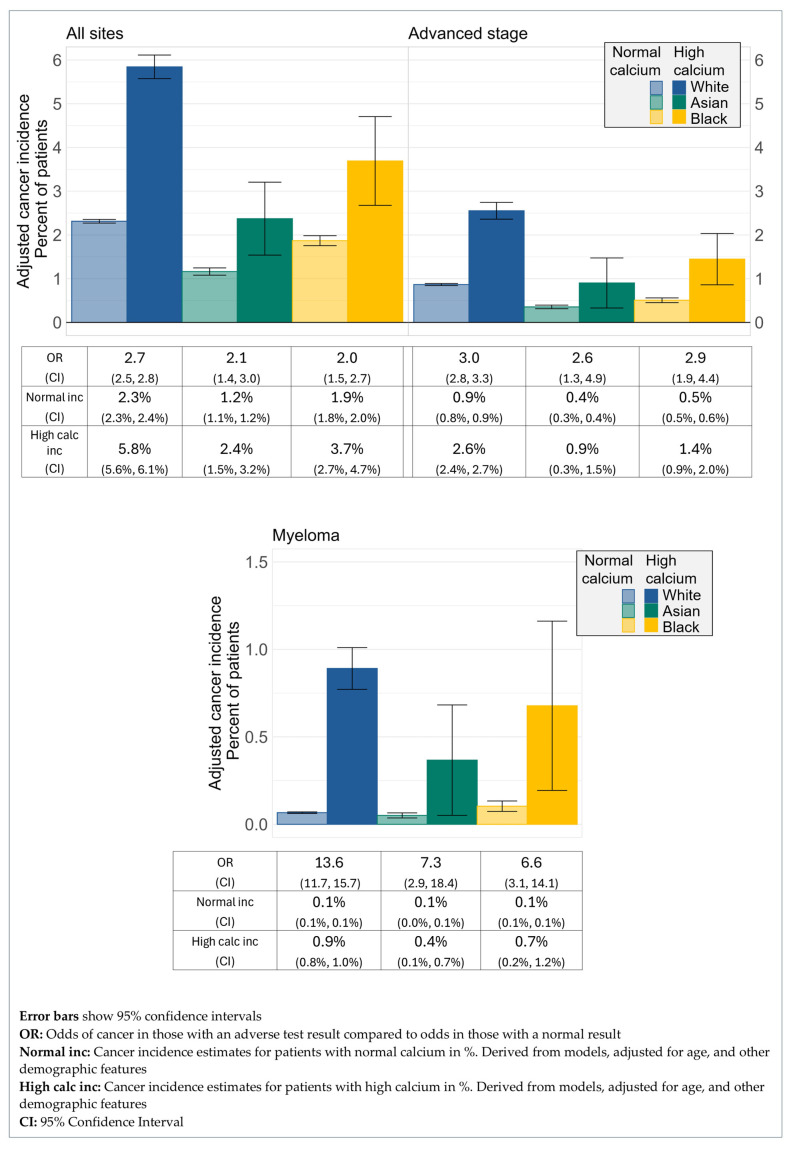
Cancer incidence by ethnicity and corrected calcium status.

The ORs for cancer detection in the year following a high calcium test result compared to a normal calcium result showed no difference between the three ethnic groups, with an OR for White patients of 2.7 (95% CI 2.5, 2.8), for Asian patients of 2.1 (95% CI 1.4, 3.0), and for Black patients of 2.0 (95% CI 1.5, 2.7) (Figure 3). For cancer diagnosed at an advanced stage, ORs again showed little difference, the OR for White patients was 3.0 (95% CI 2.8, 3.3), the OR for Asian patients was 2.6 (95% CI 1.3, 4.9), and for Black patients was 2.9 (95% CI 1.9, 4.4) (Figure 3). The OR estimate for the diagnosis of myeloma in the year following a high calcium result was highest for White patients at 13.6 (95% CI: 11.7, 15.7), but the evidence for this effect was not strong, with an OR of 7.3 (95% CI: 2.9, 18.4) for Asian patients, and an OR of 6.6 (95% CI: 3.1, 14.1) for Black patients (Figure 3).

**Figure 4:**
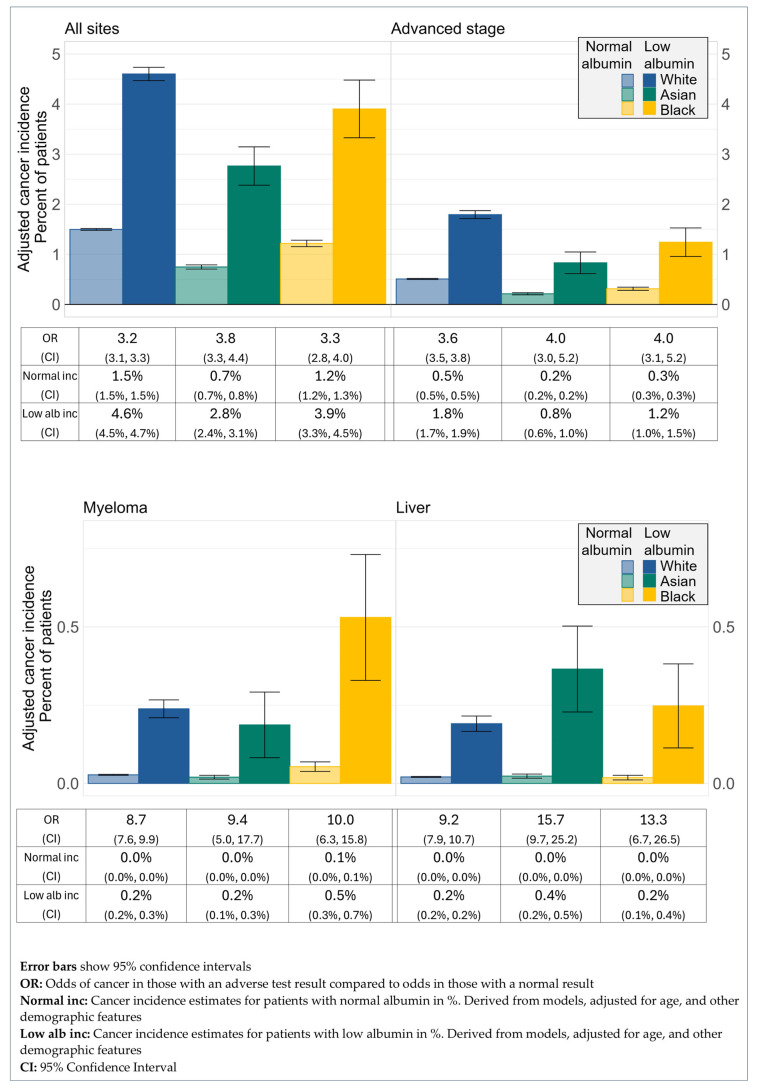
Cancer incidence by ethnicity and albumin status.

The ORs comparing cancer diagnosis for patients with low albumin compared to those with normal albumin also showed no difference between ethnic groups (Figure 4). Across all sites, the OR of cancer diagnosis within a year of the test for patients with low albumin compared to normal albumin was 3.2 for White patients (95% CI 3.1, 3.3), 3.8 for Asian patients (95% CI 3.3, 4.4), and 3.3 for Black patients (95% CI 2.8, 4.0). For those diagnosed at an advanced stage, the OR for White patients was 3.6 (95% CI 3.5, 3.8), for Asian patients 4.0 (95% CI 3.0, 5.2), and for Black patients 4.0 (95% CI 3.1, 5.2). For diagnosis of myeloma, the OR for White patients was 8.7 (95% CI 7.6, 9.9), for Asian patients 9.4 (95% CI 5.0, 17.7), and for Black patients 10.0 (95% CI 6.3, 15.8). The ORs for liver cancer diagnosis were 9.2 for White patients (95% CI 7.9, 10.7), 15.7 for Asian patients (95% CI 9.7, 25.2), and 13.3 for Black patients (95% CI 6.7, 26.5).

## 4. Discussion

### 4.1. Main Findings

Both high calcium and low albumin were predictive of cancer risk in each of the three main ethnic groups. There was little evidence of differences in cancer risk following an abnormal test result between ethnic groups.

### 4.2. Detailed Findings

Independently of blood test results, the rates of all-site cancer and cancer diagnosed at an advanced stage were highest for White patients, incidence of myeloma was highest for Black patients, and the highest liver cancer rate was seen in Asian patients. This reflects differences in the underlying cancer incidence between ethnic groups that is typically observed in UK cohorts [29] and was not examined in detail.

No ethnicity-specific differences were found in the utility of high calcium for assessment of cancer risk. Although for myeloma the point estimate of the OR was much higher for White patients than other ethnic groups, the evidence for this effect was not strong.

Similarly, the ability of low albumin to predict cancer risk was similar across the three ethnic groups studied. Although for the four analyses reported, the OR estimate for White patients was lower than those for Asian or Black patients, the number of cancer cases was not high enough to draw any meaningful conclusions from this finding.

### 4.3. Strengths and Limitations

This study was based on a very large dataset, covering approximately 20% of the UK population [30] and a time frame of eight years. Information on patient ethnicity and blood test results are routinely recorded in the dataset. The ethnic make-up of the cohort is broadly representative of the population of England and Wales [31].

Ethnicity is routinely recorded in health care records in the UK and was available for over 90% of patients in the source database. The five-category ethnicity classification (White, Asian, Black, Mixed, Other) was used to ensure that a large number of patients were available in each group, and for consistency with the UK Census groupings. The drawback of using these high-level categories is that it is not possible to investigate any effects between subgroups, such as between those in the Black African and Black Caribbean groups. In addition, the relatively low number of patients and heterogeneity within the Mixed and Other ethnic groups meant that it was not possible to assess calcium and albumin for these groups. The low cancer risk figures reported are a result of the inclusion of all patients with a blood test result, with no consideration of the indication for the test. It was not possible to infer the reason for any blood test, so only a small fraction of those included will have been carried out with a potential cancer diagnosis in mind. UK guidance only recommends the use of hypercalcaemia as a potential marker of myeloma, with no mention of albumin measurements, which may also explain the low cancer risk for patients with an abnormal test result. It is also possible that there are ethnic biases in primary care attendance and the process of blood testing, the assessment of these factors was outside of the scope of this study.

The proportion of patients with an abnormal blood result within this cohort was fairly low, at between 0.7% and 3.2% of patients, depending on the blood test and ethnic group. This explains the wide confidence intervals for some of the results, despite the large population included. An alternative approach of comparing quantiles of blood test results may have given more insight into cancer risk at different blood marker levels.

### 4.4. Comparison with Existing Literature

The association between hypercalcaemia and cancer risk, as observed in this work, is known [9,11,32], although no studies were found reporting a link with cancer stage at diagnosis, and only one study was identified which found a link with myeloma [33].

A number of studies have reported the link between low albumin and generic cancer risk [12,16,17]; however, again, no studies were identified reporting the link between low albumin and diagnosis of cancer at an advanced stage. Two studies found an association between low albumin and liver cancer [16,17], but studies reporting a link with myeloma were not found in the literature.

Very few studies were found examining either calcium or albumin, cancer incidence, and ethnicity. Yoon et al. [7] reported an inverse association between albumin levels and lung cancer risk separately by ethnic group, while Walts et al. [34] used the same cohort to identify an inverse association between albumin and colorectal cancer risk for African American women only; however, no studies were found reporting on ethnicity-specific effects for the cancer sites covered in this project.

### 4.5. Implications for Research and Practice

Unexplained hypercalcaemia and hypoalbuminaemia in primary care can be indicative of undiagnosed cancer and warrant further investigation. This study confirmed that an observation of high calcium can be indicative of myeloma, in line with current guidance suggesting that patients with hypercalcaemia should be considered for further investigations to rule out myeloma [3]. No differences between the ethnic groups were found, which is clinically useful in light of the higher incidence of myeloma in Black patients.

An observation of low albumin was found to be indicative of liver cancer risk and, to a lesser extent, myeloma. This is not represented in clinical guidance in the UK, although the link between albumin and liver disease is well known. Further research to validate these relationships would be valuable.

## 5. Conclusions

A link between high levels of calcium and myeloma incidence was found, in addition to a relationship between low albumin and liver cancer, and low albumin and myeloma. Despite modest ethnic differences in typical blood levels of these markers, little difference was found in their predictability of cancer risk by ethnicity. These findings suggest that the current method of interpreting these results without reference to patient ethnicity is acceptable.

## Figures and Tables

**Table 1:** Cohort characteristics.

Ethnicity	Number of Patients	Age	Overweight or Obese	Patients with Haemoglobinopathy	Patients In Most Deprived Category	Patients in Highest Multimorbidity Category	Patients ever Smokers	Patients Diagnosed with Any Cancer Within One Year of Test
	n (%)	Median (SD)	n (%)	n (%)	n (%)	n (%)	n (%)	n (%)
**Corrected calcium cohort**								
**White**	1,707,755 (86.3%)	62 (14.1)	583,824 (34.2%)	3744 (0.2%)	270,740 (15.9%)	445,736 (26.1%)	1,039,689 (60.9%)	42,529 (2.5%)
**Asian**	139,215 (7.0%)	53 (12.0)	53,756 (38.6%)	5352 (3.8%)	30,074 (21.6%)	26,706 (19.2%)	39,824 (28.6%)	1160 (0.8%)
**Black**	93,873 (4.7%)	51 (12.2)	43,735 (46.6%)	7111 (7.6%)	40,111 (42.7%)	14,235 (15.2%)	33,938 (36.2%)	1189 (1.3%)
**Mixed**	18,747 (0.9%)	51 (12.1)	7037 (37.5%)	873 (4.7%)	5270 (28.1%)	2830 (15.1%)	9005 (48.0%)	233 (1.2%)
**Other**	20,173 (1.0%)	51 (12.2)	7306 (36.2%)	636 (3.2%)	5854 (29.0%)	2614 (13.0%)	9182 (45.5%)	212 (1.1%)
**Total**	1,979,763	60 (14.1)	695,658 (35.1%)	17,716 (0.9%)	352,049 (17.8%)	492,121 (24.9%)	1,131,638 (57.2%)	45,323 (2.3%)

**Albumin cohort**								
**White**	4,043,319 (87.3%)	58 (13.7)	1,396,159 (34.5%)	7179 (0.2%)	650,767 (16.1%)	867,905 (21.5%)	2,412,010 (59.7%)	68,989 (1.7%)
**Asian**	308,037 (6.6%)	50 (11.6)	118,606 (38.5%)	10,042 (3.3%)	79,440 (25.8%)	45,853 (14.9%)	91,266 (29.6%)	1768 (0.6%)
**Black**	191,267 (4.1%)	49 (11.5)	89,671 (46.9%)	13,708 (7.2%)	84,935 (44.4%)	22,417 (11.7%)	69,243 (36.2%)	1699 (0.9%)
**Mixed**	43,421 (0.9%)	49 (11.2)	16,492 (38.0%)	1814 (4.2%)	12,660 (29.2%)	4986 (11.5%)	20,681 (47.6%)	363 (0.8%)
**Other**	46,812 (1.0%)	49 (11.5)	17,338 (37.0%)	1211 (2.6%)	13,670 (29.2%)	4632 (9.9%)	21,064 (45.0%)	416 (0.9%)
**Total**	4,632,856	57 (13.6)	1,638,266 (35.4%)	33,954 (0.7%)	841,472 (18.2%)	945,793 (20.4%)	2,614,264 (56.4%)	73,235 (1.6%)

**Table 2:** Blood test result distribution by ethnic group *.

Ethnicity	Median Test Result (Inter-Quartile Range)		Percent of Patients with	
	Corrected Calcium (mmol/L)	Albumin (g/L)	Hypercalcaemia	Low Albumin
**White**	2.32 (2.25, 2.40)	43 (40, 45)	1.4%	3.2%
**Asian**	2.31 (2.24, 2.39)	42 (40, 45)	1.0%	2.6%
**Black**	2.33 (2.26, 2.40)	42 (39, 45)	1.5%	3.1%
**Mixed**	2.32 (2.24, 2.39)	42 (40, 45)	1.2%	2.6%
**Other**	2.30 (2.24, 2.37)	43 (40, 45)	0.7%	2.1%

* Blood test distribution values are based on the entire eligible cohort for each blood result, including those patients with raised albumin or low corrected calcium.

## Data Availability

The data was provided under licence from CPRD; therefore, we are unable to share this dataset.

## Abbreviations

The following abbreviations are used in this manuscript:

OR	Odds ratio
CPRD	Clinical Practice Research Datalink
HES	Hospital Episode Statistics
NCRAS	National Cancer Registration and Analysis Service
BMI	Body mass index
CMS	Cambridge multimorbidity score
CI	Confidence interval

## Appendix A

**Table A1:** Calcium and albumin blood test distribution by age and sex *.

	Median Test Result (Inter-Quartile Range)		Percent of Patients with	
	Corrected Calcium (mmol/L)	Albumin (g/L)	Hypercalcaemia	Low Albumin
**Sex**				
Male	2.31 (2.24, 2.38)	43 (40, 45)	0.8%	2.5%
Female	2.33 (2.26, 2.40)	42 (39, 45)	1.8%	3.6%
**Age group**				
40–49	2.30 (2.23, 2.37)	43 (41, 46)	0.5%	1.7%
50–59	2.32 (2.25, 2.39)	43 (40, 45)	0.9%	1.8%
60–69	2.33 (2.26, 2.40)	43 (40, 45)	1.4%	2.5%
70 +	2.34 (2.27, 2.41)	41 (38, 44)	2.5%	7.1%

* Blood test distribution values are based on the entire eligible cohort for each blood result, including those patients with raised albumin or low corrected calcium.

**Table A2:** Cancer risk by blood test result including patients in the Mixed and Other groups *.

Blood Test	Cancer Site	Ethnicity	Odds Ratio (95% CI)	Incidence for Those with a Normal Test Result (95% CI)	Incidence for Those with an Abnormal Test Result (95% CI)
Corrected calcium	All cancers	White	2.7 (2.5, 2.8)	2.3% (2.3%, 2.4%)	5.8% (5.6%, 6.1%)
		Asian	2.1 (1.4, 3.0)	1.2% (1.1%, 1.2%)	2.4% (1.5%, 3.2%)
		Black	2.0 (1.5, 2.7)	1.9% (1.8%, 2.0%)	3.7% (2.7%, 4.7%)
		Mixed	2.2 (1.1, 4.5)	1.8% (1.6%, 2.1%)	3.8% (1.3%, 6.3%)
		Other	5.5 (2.9, 10.3)	1.4% (1.3%, 1.6%)	7.2% (3.2%, 11.1%)
Corrected calcium	All cancers, diagnosed at advanced stage	White	3.0 (2.8, 3.3)	0.9% (0.8%, 0.9%)	2.6% (2.4%, 2.7%)
		Asian	2.6 (1.3, 4.9)	0.4% (0.3%, 0.4%)	0.9% (0.3%, 1.5%)
		Black	2.9 (1.9, 4.4)	0.5% (0.5%, 0.6%)	1.4% (0.9%, 2.0%)
		Mixed	2.4 (0.8, 7.7)	0.6% (0.5%, 0.8%)	1.5% (−0.2%, 3.2%)
		Other	8.5 (3.8, 19.2)	0.5% (0.4%, 0.6%)	4.1% (1.1%, 7.1%)
Corrected calcium	Myeloma	White	13.6 (11.7, 15.7)	0.1% (0.1%, 0.1%)	0.9% (0.8%, 1.0%)
		Asian	7.3 (2.9, 18.4)	0.1% (0.0%, 0.1%)	0.4% (0.1%, 0.7%)
		Black	6.6 (3.1, 14.1)	0.1% (0.1%, 0.1%)	0.7% (0.2%, 1.2%)
		Mixed	4.1 (0.5, 32.0)	0.1% (0.1%, 0.2%)	0.5% (−0.5%, 1.4%)
		Other	-	0.0% (0.0%, 0.1%)	-
Albumin	All cancers	White	3.2 (3.1, 3.3)	1.5% (1.5%, 1.5%)	4.6% (4.5%, 4.7%)
		Asian	3.8 (3.3, 4.4)	0.7% (0.7%, 0.8%)	2.8% (2.4%, 3.1%)
		Black	3.3 (2.8, 4.0)	1.2% (1.2%, 1.3%)	3.9% (3.3%, 4.5%)
		Mixed	3.9 (2.8, 5.5)	1.1% (1.0%, 1.2%)	4.2% (2.9%, 5.5%)
		Other	3.2 (2.3, 4.5)	1.1% (1.0%, 1.3%)	3.5% (2.5%, 4.5%)
Albumin	All cancers, diagnosed at advanced stage	White	3.6 (3.5, 3.8)	0.5% (0.5%, 0.5%)	1.8% (1.7%, 1.9%)
		Asian	4.0 (3.0, 5.2)	0.2% (0.2%, 0.2%)	0.8% (0.6%, 1.0%)
		Black	4.0 (3.1, 5.2)	0.3% (0.3%, 0.3%)	1.2% (1.0%, 1.5%)
		Mixed	6.6 (3.9, 11.2)	0.3% (0.3%, 0.4%)	2.0% (1.1%, 2.9%)
		Other	2.8 (1.6, 4.9)	0.4% (0.3%, 0.5%)	1.2% (0.6%, 1.8%)
Albumin	Liver	White	9.2 (7.9, 10.7)	0.0% (0.0%, 0.0%)	0.2% (0.2%, 0.2%)
		Asian	15.7 (9.7, 25.2)	0.0% (0.0%, 0.0%)	0.4% (0.2%, 0.5%)
		Black	13.3 (6.7, 26.5)	0.0% (0.0%, 0.0%)	0.2% (0.1%, 0.4%)
		Mixed	9.9 (2.5, 39.4)	0.0% (0.0%, 0.0%)	0.2% (−0.1%, 0.5%)
		Other	16.0 (2.9, 89.3)	0.0% (0.0%, 0.0%)	0.2% (−0.1%, 0.4%)
Albumin	Myeloma	White	8.7 (7.6, 9.9)	0.0% (0.0%, 0.0%)	0.2% (0.2%, 0.3%)
		Asian	9.4 (5.0, 17.7)	0.0% (0.0%, 0.0%)	0.2% (0.1%, 0.3%)
		Black	10.0 (6.3, 15.8)	0.1% (0.0%, 0.1%)	0.5% (0.3%, 0.7%)
		Mixed	23.8 (5.3, 107.8)	0.0% (0.0%, 0.0%)	0.3% (−0.0%, 0.7%)
		Other	33.4 (9.6, 116.6)	0.0% (0.0%, 0.0%)	0.5% (0.1%, 0.9%)

* Cancer incidence estimates were derived from models, adjusted for age category, sex, year of index blood test, deprivation category, multimorbidity category, BMI category, smoking status, and haemoglobinopathy indicator.
